# Exercise-induced sudden cardiac death is caused by mitochondrio-nuclear translocation of AIF

**DOI:** 10.1038/s41419-021-03677-w

**Published:** 2021-04-09

**Authors:** Mahmoud Abdellatif, Guido Kroemer

**Affiliations:** 1 Department of Cardiology, Medical University of Graz, Graz, 8036 Austria; 2 Metabolomics and Cell Biology Platforms, Institut Gustave Roussy, Villejuif, 94805 France; 3 Centre de Recherche des Cordeliers, Equipe labellisée par la Ligue contre le cancer, Université de Paris, Sorbonne Université, INSERM U1138, Institut Universitaire de France, Paris, 75006 France; 4 Pôle de Biologie, Hôpital Européen Georges Pompidou, AP-HP, Paris, 7015 France; 5 Suzhou Institute for Systems Medicine, Chinese Academy of Medical Sciences, Suzhou, 215000 China; 6 Karolinska Institute, Department of Women’s and Children’s Health, Karolinska University Hospital, Solna, 17164 Sweden

**Keywords:** Apoptosis, Necroptosis, Arrhythmias

Irrespective of the underlying risk factors, myocyte cell death is a common feature in cardiac disorders. In particular, arrhythmogenic cardiomyopathies (ACMs) are characterized by an increased incidence of apoptotic and necrotic cardiac myocytes. Since cardiac myocytes are predominantly post-mitotic^1^, they are typically replaced by fibrous tissue, which further interferes with the electrophysiology of the heart, adding to the disruption of the cardiac conduction system and, thus, ACM progression. Notably, ACM patients exhibit poor prognosis and increased risk of sudden cardiac death in association with exercise. Thus, ACM is one of the rare conditions in which exercise is detrimental rather than beneficial for organismal health^2^. However, the underlying mechanisms of this intriguing, but well-established, observation remain poorly understood. In their recent study, Chelko et al. used homozygous desmoglein-2 mutant mice (*Dsg2*^*mut/mut*^), which exhibit multiple features of human ACM, to elegantly unveil the molecular underpinnings of the exercise-induced myocyte cell death in ACM^3^. At the core of their proposed mechanistic cascade lies the apoptosis-inducing factor (AIF), a pro-apoptotic signaling molecule that is normally located in the mitochondrial intermembrane space. However, in response to apoptotic cues, AIF moves to the nucleus, thereby triggering DNA fragmentation and cell death^4,5^.

Chelko et al. found that almost half of *Dsg2*^*mut/mut*^ mice die before finishing an eleven-week protocol of endurance swimming, as compared to less than 10% in the case of WT controls. Trained *Dsg2*^*mut/mut*^ mice that did not die suffered from severe biventricular dilation and systolic dysfunction, and showed electrocardiograghic signs of impaired cardiac depolarization and repolarization. Concurrently, *Dsg2*^*mut/mut*^ hearts exhibited exaggerated fibrosis, immune cell infiltration, and increased abundance of necrotic cardiomyocytes, as determined by high-mobility-group box-1 immunostaining^3^. The authors went on to show that *Dsg2*^*mut/mut*^ hearts overexpress the Ca^2+^-activated cysteine protease calpain 1 (CAPN1), which was mainly localized at mitochondria. The cytosol-to-mitochondria translocation of CAPN1 appeared to be a prerequisite for its Ca^2+^-dependent activation. The authors also attributed such increases in CAPN1 activity to the reduced abundance of its endogenous inhibitor, calpastatin (CAST) in *Dsg2*^*mut/mut*^ hearts. In support of this notion, CAST overexpression or treatment with the CAPN1 inhibitor, calpeptin, delayed myocyte cell death induced by Ca^2+^ overload or CAPN1 in vitro^3^.

In an effort to determine the mitochondrial effectors responsible for cardiac myocyte necrosis in *Dsg2*^*mut/mut*^ mice, the authors examined key mediators of mitochondrial cell death. Specifically, they examined cytochrome C and apoptosis-inducing factor-1, mitochondrial (AIFM1, best known as AIF). While cytochrome C was not changed in *Dsg2*^*mut/mut*^ hearts, AIF exhibited significant calpain-mediated truncation (which removes a hydrophobic domain from AIF that retains it at the mitochondrial inner membrane), mitochondrio-nuclear translocation and chromatin binding in response to exercise. Importantly, Chelko et al. showed that truncated AIF undergoes nuclear translocation not only in the myocardium of exercised *Dsg2*^*mut/mut*^ mice, but also in ventricular samples of patients with ACM. In an interesting twist of their study, the authors demonstrated that a major proportion of cleaved AIF in *Dsg2*^*mut/mut*^ mice is oxidized, an effect which they attributed to exaggerated exercise-triggered oxidative stress due to an insufficient mitochondrial thioredoxin-2 ROS buffering system. In turn, the degree of AIF oxidation was found to increase its DNA binding ability, thus facilitating AIF-mediated chromatinolysis.

Finally, in a series of exhaustive experiments the authors could show that truncated AIF causally underlies ACM-associated myocyte cell death. First, they showed that HSP70, an endogenous inhibitor of AIF, was reduced both in *Dsg2*^*mut/mut*^ hearts and stem cell-derived cardiomyocytes that were subjected to sustained beta-adrenergic stimulation and Ca^2+^ overload to mimic exercise. Along similar lines, both *Dsg2*^*mut/mut*^ myocardia and stem cell-derived cardiomyocytes exhibited higher abundance of the AIF nuclear chaperone, peptidyl-prolyl cis-trans isomerase (PPIA or cyclophilin-A), which was bound to AIF and co-migrated with it to the nucleus. Next, an AIF mimetic peptide, fused to the cell-penetrating HIV transactivator of transcription (TAT), was used to test whether blocking the interaction between AIF and PPIA would prevent cell death induced by chronic beta-adrenergic stimulation and Ca^2+^ overload in *Dsg2*^*mut/mut*^ stem cell-derived cardiomyocytes. AIF-TAT peptide reduced the formation and nuclear migration of PPIA–AIF complexes in these cells, thereby plummeting myocyte apoptotic death. Interestingly, AIF-TAT treatment also attenuated signs of necrotic cell death such as nuclear enlargement, loss of cardiac troponin striations, and cell membrane swelling, all of which were evident in untreated ACM myocytes. These results suggest that preventing the interaction between AIF and PPIA effectively protects from both apoptosis and necrosis.

The authors are to be commended for their diligent efforts to meticulously dissect the underlying mechanisms of exercise-induced cardiomyocyte cell death in ACM (Fig. 1). Future studies must test whether pharmacological targeting of AIF, for instance by inhibiting its oxidation, truncation, interaction with PPIA, or translocation to the nucleus, also protects against ACM-related sudden cardiac death in vivo. Thus, it will be interesting to see whether genetic inactivation of *PPIA* can correct the phenotype of *Dsg2*^*mut/mut*^ mice, knowing that, in PPIA knockout mice, reduced mitochondrio-nuclear translocation of AIF exerts neuroprotective effects against cerebral injury induced by hypoxia-ischemia^6^. It will also be interesting to examine whether cardiac non-myocytes, especially neurons and resident macrophages, which contribute to cardiac electrical conduction^7^, are subjected to similar pro-apoptotic AIF actions in response to exercise. Furthermore, future research must determine whether other AIF functions, beyond its pro-apoptotic action, are involved in ACM. For instance, AIF is implicated in the biogenesis of mitochondrial respiratory chain complexes, meaning that it is required for oxidative phosphorylation^8^. Considering the acute nature of sudden cardiac death, impaired mitochondrial function might be an added mechanism through which cleaved AIF contributes to ACM. In favor of this conjecture, Cheiko et al. reported that *Dsg2*^*mut/mut*^ cardiac myocytes exhibit a reduced mitochondrial membrane potential, limited anti-oxidative capacity and increased oxidative stress^3^. Thus, it is tempting to speculate that *Dsg2*^*mut/mut*^ hearts might also suffer from a mitochondrial bioenergetic crisis, which might promote cardiac dysfunction and mortality upon exercise. Indeed, mitochondrial energy metabolism is increasingly implicated in various forms of cardiomyopathies, including heart failure^9,10^. Thus, future studies should examine whether improving mitochondrial function improves ACM and reduces exercise-induced sudden death of ACM patients. Indeed, mounting evidence suggests that reduced mitochondrial ROS and Ca^2+^ accumulation^11,12^, as well as the promotion of mitochondrial quality control mechanisms, like autophagy/mitophagy, are associated with salutary cardiac effects and protection against a wide range of cardiac diseases^13–15^. Finally, although the study by Chelko et al. provides initial evidence for a role of AIF in human ACM, further clinical studies will be needed to corroborate the reported mechanisms in humans. To achieve this goal, larger patient cohorts with pre-mortem diagnosis of ACM, and detailed history on the cause of death and exercise training will be necessary. For this, cardiac samples, preferably collected prospectively from live ACM patient (rather than at autopsy), would be of great utility for further elucidating the pathogenesis of the disease.

**Fig. 1:**
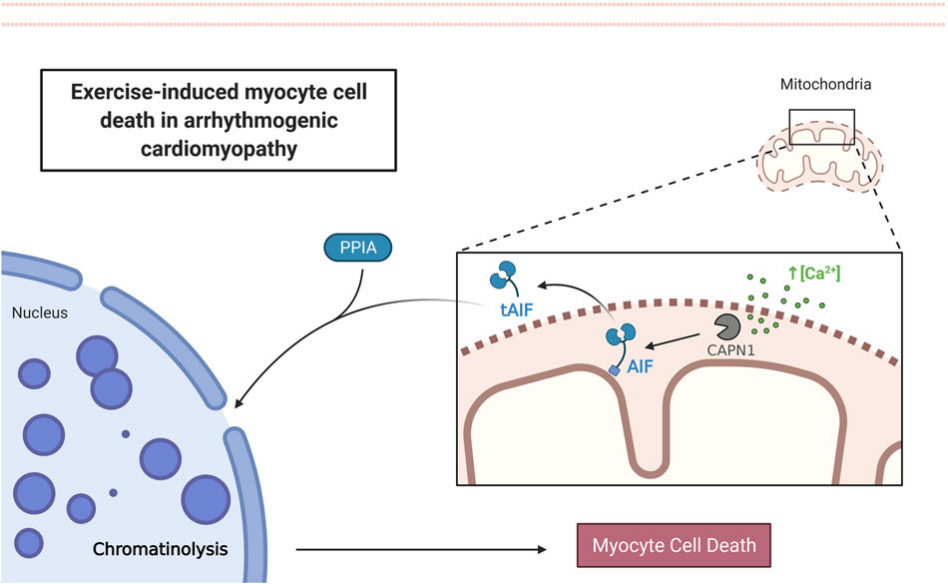
Mitochondrio-nuclear translocation of AIF underlies exercise-induced myocyte cell death in arrhythmogenic cardiomyopathy (ACM). Simplified schematic depiction of the underlying mechanistic events leading to myocyte cell death in ACM. Briefly, calcium overload activates cysteine protease calpain-1 (CAPN1), which cleaves apoptosis-induced factor (AIF) leading to its release from the mitochondrial intermembrane space. Truncated AIF (tAIF) then migrates, in conjunction with its chaperone, peptidyl-prolyl cis-trans isomerase (PPIA, best known as cyclophilin A), to the nucleus where it induces DNA fragmentation and subsequent cell death. (created with biorender.com).

In conclusion, the findings reported by Chelko et al. establish the CAPN1-PPIA-AIF axis as a firm therapeutic target candidate for treating ACM and for avoiding exercise-induced sudden cardiac death.

## References

[1] Hauck, L., Dadson, K., Chauhan, S., Grothe, D. & Billia, F. Inhibiting the Pkm2/b-catenin axis drives in vivo replication of adult cardiomyocytes following experimental MI. *Cell Death Differ.*10.1038/s41418-020-00669-9 (2020). 10.1038/s41418-020-00669-9 PMC8027412 33288902

[2] López-Otín C, Kroemer G (2021). Hallmarks of health. Cell.

[3] Chelko S. P. et al. Exercise triggers CAPN1-mediated AIF truncation, inducing myocyte cell death in arrhythmogenic cardiomyopathy. *Sci Transl Med* **13**. 10.1126/scitranslmed.abf0891 (2021) 10.1126/scitranslmed.abf0891 PMC8936193 33597260

[4] Susin SA (1999). Molecular characterization of mitochondrial apoptosis-inducing factor. Nature.

[5] Vahsen N (2006). Physical interaction of apoptosis-inducing factor with DNA and RNA. Oncogene.

[6] Zhu C (2007). Cyclophilin A participates in the nuclear translocation of apoptosis-inducing factor in neurons after cerebral hypoxia-ischemia. J. Exp. Med..

[7] Hulsmans M (2017). Macrophages facilitate electrical conduction in the heart. Cell.

[8] Hangen E (2015). Interaction between AIF and CHCHD4 regulates respiratory chain biogenesis. Mol. Cell.

[9] Fernandez-Caggiano, M. & Eaton P. Heart failure-emerging roles for the mitochondrial pyruvate carrier. *Cell Death Differ*10.1038/s41418-020-00729-0 (2021) 10.1038/s41418-020-00729-0 PMC8027425 33473180

[10] Zimmermann A, Madreiter-Sokolowski C, Stryeck S, Abdellatif M (2021). Targeting the mitochondria-proteostasis axis to delay aging. Front Cell Dev. Biol..

[11] Santin Y (2020). Mitochondrial 4-HNE derived from MAO-A promotes mitoCa2+ overload in chronic postischemic cardiac remodeling. Cell Death Differ..

[12] Xie Y (2020). The proteasome activator REGγ accelerates cardiac hypertrophy by declining PP2Acα-SOD2 pathway. Cell Death Differ..

[13] Yang RM (2019). TAMM41 is required for heart valve differentiation via regulation of PINK-PARK2 dependent mitophagy. Cell Death Differ..

[14] Fan F (2020). Deletion of heat shock protein 60 in adult mouse cardiomyocytes perturbs mitochondrial protein homeostasis and causes heart failure. Cell Death Differ..

[15] Abdellatif M, Ljubojevic-Holzer S, Madeo F, Sedej S (2020). Autophagy in cardiovascular health and disease. Prog. Mol. Biol. Transl. Sci..

